# Supplementary material for: Are carbon emissions trading and green financial instruments synergistic? -Comprehensive quantitative research based on content analysis
Source: PLoS One. 2024 Mar 7;19(3):e0298601. doi: 10.1371/journal.pone.0298601 (PMC10919601; doi:10.1371/journal.pone.0298601)
Supplement: S1 Data — (ZIP) [file pone.0298601.s001.zip › Data/地级市Z_F工作报告.zip!/░▓╗╒/╬▀║■╩╨/└┤╫╘╣1⁄2╓┌║┼í╛┬φ┐╦╩2╛▌═°í┐_2008.docx]

——2008年 1月5日在芜湖市第十四届人民代表大会第一次会议上

市长 陈树隆

各位代表：

现在，我代表市人民政府，向大会作政府工作报告，请予审议，并请市政协委员和其他列席人员提出意见。

一、过去五年的工作回顾

过去五年是不平凡的五年。五年来，在中共芜湖市委的坚强领导下，全市人民认真贯彻落实科学发展观，牢牢把握发展机遇，积极应对环境变化，开拓创新，锐意进取，扎实推进改革开放和现代化建设，经济社会保持了又好又快的发展势头。

（一）经济持续快速增长，综合实力明显提升

经过五年发展，全市生产总值由254亿元增加到580亿元（预计数，下同），年均增长15.1%；人均地区生产总值超过3300美元，年均增长14.3%。财政收入由36.2亿元增加到105亿元，年均增长23.8%。社会消费品零售总额由80.1亿元增加到164亿元，年均增长15.4%。金融机构贷款余额由184亿元增加到452亿元，年均增长19.7%。五年累计完成固定资产投资1251亿元，年均增长34.5%。建成了奇瑞汽车改扩建、海螺水泥熟料、芜宣高速、芜马高速、沿江高速芜铜段、华强旅游城方特欢乐世界等一批事关全局发展的重大项目。产业结构进一步优化，一、二、三次产业比重由9.6：49.6：40.8调整为5.7：58.1：36.2。都市型现代农业快速发展，服务业发展水平得到提升，工业经济主导地位进一步确立。

（二）经济增长质量不断提高，经济发展方式明显转变

产业集群化趋势明显，企业核心竞争力普遍增强。2007年汽车及零部件、材料、电子电器产业完成产值740亿元，对全市规模以上工业增长的贡献率达81%。新建了5个省级开发区。2007年园区工业实现总产值657亿元，占全市规模以上工业总产值的68.5%。五年新增省级以上高新技术企业105家，高新技术产业增加值占全市工业增加值的比重由18%上升到44%。奇瑞成为中国汽车工业史上第一个乘用车产销量突破百万辆的自主品牌。全市35%的规模以上工业企业建立了研发机构。五年建成省级以上工程技术研究中心5家、企业技术中心22家、重点实验室3个，获得驰名商标2个。科技进步对经济增长的贡献率达57%。查处关闭了一批违法排污企业，建设了一批污水、垃圾处理设施，加强了生态环境建设，实现了工业污染源全面达标排放，城市空气、供水质量位居全国前列，经济增长向低能耗、低排放、高产出、高效益加快转变。

（三）统筹城乡发展格局初步形成，城乡面貌发生明显变化

按照城乡产业发展、规划管理、基础设施、社会事业、就业和社会保障、生态环境建设一体化的要求，着力推进社会主义新农村建设。建成市县快速通道芜繁路、芜屯路，建设农村公路2200公里。完成了长江干堤除险加固工程。实施了万亩以上圩口堤防达标工程，累计完成投资2.3亿元。开展全国农村商品流通改革和市场建设试点。改进农村公共服务，加快了村庄整治步伐。对城市行政区划进行了重大调整，市区面积由230平方公里扩大到720平方公里，建成区面积由68平方公里扩展到117平方公里。新建、改造了九华北路、银湖路等16条城市主干道，实施了滨江公园、九莲塘公园等一批城建项目。五年城市基础设施建设共投入288亿元，是前五年的3倍。

（四）改革开放不断推进，发展动力明显增强

农村税费改革全面完成，综合改革不断深化，农村基层工作新机制基本建立。深化市、区财政体制改革，推进部门预算、国库集中支付、政府采购制度改革，构建了公共财政体系的基本框架。完善国有资产管理体制，市级国有经营性资产由五年前的40亿元增加到131亿元。基本完成国有和集体工业企业产权制度改革。民营经济发展迅速，五年新增私营企业8700户，非公有制经济比重达60%以上。深化农村信用社改革，成立了中部地区首家农村商业银行——芜湖扬子农村商业银行，初步建立了中小企业信用担保体系。五年实际利用外资14.4亿美元、省外资金762亿元，年均分别增长30.9%、43.3%。2007年进出口总额达16.5亿美元，年均增长42.9%。港口年吞吐量由1730万吨增加到4600万吨，年均增长21.6%。经济外向度由9%提升到20%以上。

（五）民生工作扎实开展，人民生活明显改善

五年累计新增就业岗位23.9万个，城镇登记失业率控制在4%以内。社会保障体系框架基本建立。全市城镇职工养老保险参保30.3万人，年均增加1.5万人，企业退休人员养老金人均每月由492元提高到868元。失业保险参保22万人，失业金每人每月由223元提高到340元。城镇职工医疗保险参保32.8万人，医药费个人负担比例由33.5%下降到19.9%。建立了工伤、生育保险制度，参保人员分别达16万人、10.1万人。城乡居民最低生活保障基本实现了应保尽保，城市、农村低保标准分别由每月192元、60元提高到252元、80元。城市居民人均住房使用面积和农村居民人均生活用房面积由15.6平方米、27.2平方米分别提高到18.4平方米、34平方米。全市私人汽车保有量由4700辆增加到18900辆。城市居民人均可支配收入由6493元增加到13000元，农村居民人均纯收入从2853元增加到5050元，年均分别增长14.9%和12.1%。

（六）各项事业全面进步，社会建设明显加强

组织开展了“创新文化”、“坚持科学发展、打造五大优势”等解放思想大讨论，推进“诚信芜湖”建设，群众性精神文明创建活动取得明显成效。率先实施了农村义务教育管理体制改革，大力推进中小学均衡发展，中小学布局调整取得积极进展，“两基”成果得到巩固，职业教育规模进一步扩大。成立了芜湖信息技术职业学院、安徽机电职业技术学院、安徽商贸职业技术学院、芜湖技师学院。高教园区基本建成。芜湖职业技术学院进入国家重点建设示范高职院校行列。深化文化体制改革，实施了科技馆等一批重点项目，新增一批重点文物保护单位，文化事业和文化产业进一步发展。深化疾病预防和卫生监督体制改革，加强公共卫生基础设施建设，完善了城乡卫生服务网络。人口和计划生育综合改革扎实推进，低生育水平保持稳定。完成了第六届村委会、第五届居委会换届选举，社区建设进一步加强。开展了第一次经济普查、第二次农业普查。加强国防后备力量和人防建设。我市被命名为全国双拥模范城。档案、地方志、民族、宗教、气象、外事、侨务、对台、妇女儿童、老龄、残疾人等事业取得新进步。

（七）政府自身建设不断加强，依法行政水平明显提高

认真贯彻落实《全面推进依法行政实施纲要》。自觉接受人大及其常委会法律监督、工作监督和政协民主监督，广泛听取和采纳各界人士的意见和建议，五年共办理人大代表议案和建议417件，政协建议案、委员提案和建议2113件，办复率达100%。完善用人、用钱、用地、用权制度，推进“四用”制度向县区、镇村延伸，向其它领域拓展，政府工作效能进一步提高。对行政审批和行政事业性收费项目进行了多次清理，减少审批项目40%。完善行政服务中心运行机制，单个项目平均办结周期由15.1个工作日减少到2.3个工作日。在全省率先成立市级招标采购中心，累计办理招标项目4700多个。建立健全政府新闻发布制度，加强电子政务建设，开通了“市民心声”网站，增强了政府工作透明度和公众参与度。加大对商业贿赂等损害群众利益不正之风的治理力度，严肃查处了一批案件。

2007年是本届政府任期的最后一年。一年来，我们扎实推进各项工作，完成了市十三届人大七次会议确定的主要目标任务，全市发展呈现出增长较快、结构优化、效益提高、民生改善的良好态势。全市生产总值净增100亿元，财政收入突破100亿元，固定资产投资净增140亿元，工业企业总产值突破1000亿元。预计，全市实现生产总值比上年增长17%；财政收入增长27%，其中地方财政收入增长23%；全社会固定资产投资增长49%；社会消费品零售总额增长17.5%；进出口总额增长89.7%；城市居民人均可支配收入、农民人均纯收入分别增长20%和11.9%；人口出生率控制在9‰以内。重点抓了以下七个方面的

一是大力推进新农村建设。全年市级调度资金21.8亿元，支持县区发展。认真落实支农惠农政策，积极实施各项富民工程，都市型现代农业快速发展。全市农林牧渔业总产值56.5亿元，增长3.8%。粮食总产量达83万吨，创历史最好水平。新增省级农业产业化龙头企业5家、无公害农产品认证13个、绿色食品认证18个、有机食品1个。发展农民专业合作经济组织218个，带动农户10.4万户。启动了集体林权制度改革。加强了长江及内河河道黄砂管理。继续推进公共设施和公共服务向农村延伸覆盖。制定了新型农村社区居民点布点规划。芜南路快速通道三期和芜南供水项目已进场施工。万亩以上圩口堤防达标工程完成投资5000万元。新建水泥路406公里，在全省率先实现了村村通水泥路目标。全市村通公交班车率达86%。加快三县天然气工程建设，推进农村改水改厕工作，农村生产生活条件进一步改善。

二是大力推动工业经济发展。全市规模以上工业实现增加值260亿元，增长 24%。一批骨干企业快速发展，年销售收入超10亿元企业达13户，增加3户；全市规模以上工业企业达874户，增加126户。规模以上企业实现利税91.5亿元，增长25%。全年完成工业投资220亿元，增长78.1%。奇瑞轿车三厂、芜湖海螺水泥二期等项目建成投产。华电芜湖电厂一期、响水涧抽水蓄能电站、新兴铸管改造等项目建设加快。争取国家、省科技计划项目63项。新认定国家、省级高新技术企业77家。获中国名牌1个、国家免检产品4个、国家和省科技奖9项。申请专利1500件，授权专利700件。编制了循环经济发展规划，大力推进节能减排，一批企业节能技改和资源综合利用项目加快推进。

三是大力发展服务业。制定了促进服务业发展的政策措施。汽车滚装码头等物流项目建设进展顺利。联盛商业广场等一批商业设施项目建设快速推进。各类中介组织发展较快，国际商会会员达206家。积极改善金融生态环境，全年实现融资总额658.9亿元，比年初增加116.7亿元，其中域外金融机构对我市贷款余额达80.6亿元。设立典当、担保机构12家。典当贷款和担保贷款全市全年累计达17.7亿元，比上年增加3.6亿元。编制了全市旅游发展规划，申报了一批4A景区。成功举办了16个专业会展，旅博会、青博会、汽博会被长三角会展城市联盟评为优质会展。

四是大力推进改革开放。妥善处理国企改制遗留问题，对芜湖造船厂等企业实施了资产重组。精诚铜业成功上市。海螺水泥实现增发。市建投公司发行长期债券10亿元。完成了党政机关资产、资金清理集中工作，市直机关撤销帐户489个，清理集中资金7.8亿元。启动了事业单位分类改革。全年实际利用外资4.6亿美元，增长34.5 %，其中外商直接投资4.4亿美元，增长152.6%；实际利用省外资金210亿元，增长47%。欧尚、台达、德尔福、江森、信义集团等一批大企业来芜发展。

五是大力加强城市规划、建设、管理工作。编制了城市总体规划。启动了商务文化中心建设。完成了东一路、东四大道、扁担河大桥建设工程。三环路、赤铸山东路、神山东路、神山南路、滨江南路一期、弋江路改扩建等工程正在实施。房地产市场监管得到加强，全年共推出经营性用地5800亩，新开工建设商品房336万平方米，限价房30万平方米，为遏制房价过快上涨打下了基础。大力开展全市拆违工作，取得了阶段性成果。开展市区户外广告位有偿出让，加强城市管理信息系统建设，管理水平进一步提高。

六是大力实施民生工程。去年投入7.8亿元，其中市级投入4.2亿元，完成了23项民生工程年度主要目标任务。完善了城乡低保制度，保障了8.5万人的基本生活。提高了农村“五保户”生活水平，集中供养和分散供养标准年人均比上年提高400元。建立健全城镇养老保障体系，实现了城镇养老保险政策全覆盖。完善农村部分计划生育家庭奖励扶助制度，奖励扶助2744人。全面实施新型农村合作医疗制度，参合农民124.2万人，参合率达87.6%。建立了城镇居民基本医疗保障制度，全年参保26.5万人。提高了救济对象大病医疗救助水平，共救助5.7万人次。推行了高危行业职工工伤保险制度，实现了高危行业工伤保险全覆盖。建立重大传染病病人医疗救治和生活救助保障机制，对艾滋病、结核病、血吸虫病病人实行了及时救治。启动惠民医疗工程，减免服务对象治疗费用。加强农村和社区卫生服务体系建设，建成乡镇卫生院13所、社区卫生服务机构100个。健全城乡就业和社会保障服务体系，全市所有镇（街道）、社区和村均设立了服务机构。强力推进签约参保，全年新增城镇职工养老保险2.3万人，医疗保险3.5万人，工伤保险2.8万人，生育保险4.8万人，新签劳动合同1.8万人。全面实施城乡义务教育经费保障机制改革，免除了义务教育阶段学生学杂费，补助了贫困寄宿生生活费，免费提供贫困生教科书。改造农村中小学危房2.8万平方米。解决了农村11万人的饮水安全问题。深入开展市区环境综合整治，改造背街背巷130条，新建旅游公厕10座，城南污水处理厂、朱家桥污水处理厂二期工程开工建设。完成了保兴垾综合整治年度目标任务。加快廉租住房建设，竣工廉租房2万平方米，1090户困难家庭获得实物配租、租金减免和租金补贴。实施食品药品安全放心工程，建立了重点食品市场准入和检测评价制度，农村药品供应网和监督网覆盖率均达90%。推进平安芜湖建设，完成了畅通工程、科技强警等项目建设任务。实施文化惠民工程，丰富了人民群众精神文化生活。

七是大力发展各项社会事业。去年市级对社会事业的投入达7.7亿元，同比增长35.1%。文明创建活动取得成效。成功举办了“倾国倾城城市日”等活动，城市知名度和美誉度进一步提高。完善中小学布点规划，加强了薄弱学校建设。巩固“普及九年义务教育”成果，改革中考中招制度，启动高中阶段教育普及工程，高中毛入学率达62%。完成了十二中新校区建设和聋哑职业学校搬迁。市二院和中医院进入三甲医院行列。编制了芜湖市文化发展规划。出台了扶持动漫产业发展政策，国家动漫产业发展基地正式挂牌。首批6个民间收藏馆正式授牌。成功举办了安徽省第五届农运会。加大耕地保护和土地开发复垦整理力度，节约集约使用土地。健全了政府应急管理体系，加强了安全生产监管。高度重视信访工作。扎实推进社会治安综合治理，严厉打击刑事犯罪和经济犯罪，刑事案件持续下降，社会和谐稳定。

五年来的实践使我们深切体会到，要长期保持经济社会又好又快发展，必须坚持科学发展，切实转变经济发展方式，努力实现速度质量效益相协调，消费投资出口相协调，人口资源环境相协调，改革发展稳定相协调；必须着力打造优势产业，坚持有限目标，有所为，有所不为，培育一批具有核心竞争力的优势骨干企业，推动集群化发展；必须坚持城乡统筹，以工促农，以城带乡，推动组团式发展，提升城市能级，增强可持续发展能力；必须深化改革开放，不断创新体制机制，为加快发展提供不竭动力；必须坚持以人为本，着力解决人民群众最关心、最直接、最现实的利益问题，让广大人民群众共享改革发展成果，建设和谐芜湖；必须弘扬创新文化，崇尚创新，宽容失败，支持冒险，鼓励冒尖，兴起并长期保持创新创业的社会热潮，使创新意识、创新精神、创新力量贯穿经济社会发展全过程。

在看到成绩的同时，我们也清醒地认识到，经济总量不够大、结构不够优、综合竞争力不够强仍是我市经济社会发展的突出矛盾，与全市人民率先发展的要求相比，我们的工作还有不小差距，主要是：自主创新能力建设需要进一步加强，节能减排任务还很艰巨；城市交通功能还不够完善，建设力度还需进一步加大；制约经济社会发展的体制性障碍还未有效破除，开放型经济发展水平还有待进一步提高；社会事业发展还显不足，社会保障体系还有待完善；农民持续增收难度增加，部分低收入群众生活还比较困难；房价上涨过快，势头没有得到有效遏制；少数政府工作人员责任意识淡薄，政府效能建设有待进一步强化。我们将高度重视这些问题，采取有效措施，着力加以解决。

总体上看，过去的五年，是城市综合经济实力大幅提升的五年，是各项社会事业全面进步的五年，是人民群众得到更多实惠的五年，是科学发展、和谐发展的五年，是芜湖发展最好最快的时期之一，全市经济社会进入了厚积薄发、加速发展的新阶段。我们遇到的困难比预料的大，取得的成绩比预期的好。面对“非典”、禽流感等灾害威胁，面对宏观经济环境偏紧、社会转型矛盾增多的复杂局面，市政府紧紧依靠全市人民，攻坚克难，奋力拼搏，圆满完成了本届政府的目标任务。五年的发展进步，是中共芜湖市委正确领导的结果，是市人大、市政协关心支持的结果，是全市上下团结一致、共同努力的结果。在此，我谨代表市人民政府，向全体人大代表、政协委员，向全市广大工人、农民、知识分子、干部、驻芜解放军指战员、武警官兵和政法干警，向各民主党派、各人民团体和社会各界人士，向在我市创业的投资者、建设者和海内外友好人士，表示衷心的感谢和崇高的敬意！

二、今后五年全面建设小康社会的目标任务

当前，我们正处于全面建设小康社会的关键时期。我们必须全面贯彻党的十七大精神，适应宏观环境的新变化，顺应人民群众过上更好生活的新期待，进一步增强危机感、紧迫感和责任感，千方百计抢抓机遇，积极主动迎接挑战，以全球的视野规划未来蓝图，以敢为人先的气魄推动改革发展，构筑经济社会发展新优势，开启更好更快发展新征程，再创改革开放新辉煌，谱写科学发展、和谐发展、率先发展新篇章。

未来五年发展的主要目标是：全面实现“十一五”规划目标，2012年，全市实现生产总值1400亿元，年均增长15%，人均地区生产总值再翻一番。实现财政收入220亿元，其中地方财政收入88亿元，年均分别增长16%。转变经济发展方式取得重大进展，万元生产总值能耗比2007年下降20%，二氧化硫、化学需氧量排放分别下降3%、11.4%。城乡一体化发展取得明显进展，城市功能进一步完善，城镇化率达67%。法治政府建设取得新成效，政府提供公共服务的能力明显增强。社会就业比较充分，人民生活水平显著提升，城市居民人均可支配收入、农民人均纯收入分别达到20900元、7080元，年均分别增长10%、7%。覆盖城乡的公共服务体系和社会保障体系基本建立，人人共享发展成果的和谐社会格局逐步形成。较为完善的文化服务体系基本建立，文化产业增加值占地区生产总值的比重达4%以上，市民文明素质明显提高。未来五年发展的主要任务是：

（一）大力打造优势产业，建设具有国际竞争力的先进制造业基地

着力调整优化产业结构，实现高效益、高质量、高速度、生态化、集群化发展。继续壮大支柱产业，形成一批具有核心竞争力的骨干龙头企业，把奇瑞、海螺培育成具有国际影响力的跨国公司，打造国内具有重要影响的汽车产业集群、电子电器产业集群和材料产业基地。大力发展装备制造业，形成新的支柱产业。运用信息技术、先进适用技术改造提升传统产业，加快电能、造船、制药、卷烟、食品加工、纺织服装等产业发展步伐。坚持产业特色，推进工业向国家级和省级开发区集中，提高产业集聚度。完善商贸流通市场布局，培育一批具有较强市场竞争力的物流企业，建设有重要影响的区域物流中心。大力发展设施农业、高效农业、生态农业，形成都市型现代农业特色块状经济。通过五年培育，形成年销售收入100亿元以上的企业7家，10亿元以上的企业40家。争取20家以上的企业通过上市做大做强，形成上市公司芜湖板块。大力推进自主创新，完善政策激励机制，优化市场环境，形成以企业为主体、市场为导向、产学研相结合的区域创新体系。实施质量振兴计划，培育一批名牌产品。加强能源资源节约和生态环境保护，降低发展的资源成本和环境成本，增强可持续发展能力。

（二）加快城乡统筹发展步伐，加速形成组团式发展格局

将城市核心区建成商务文化中心区，将城北组团区建成先进制造产业区，将城东组团区建成以现代工业、居住为主导的综合型新城区，将城南组团区建成以文化、科教、高新技术产业为主导的生态型城区，将三山组团区建成以临港工业、绿色食品加工为主导产业的综合型新城区。加快湾沚、繁阳、籍山三个组团建设步伐，拉开城市框架，加强基础设施建设，完善城市功能，发展特色产业，提高承载力、集聚力和辐射力。加快基础设施和公共服务向农村延伸覆盖，推进城乡道路、供水、供电、供气、公交、污水和垃圾处理等公用设施建设，实施村庄整治，改善农村生产生活条件，使现有自然村落逐步向布局合理、设施完善、规模适当的新型社区转变。加快交通基础设施建设，建成芜雁高速、铜南宣高速，推进长江公路二桥、徐福高速芜湖段、淮南铁路复线、宁宜城际铁路、芜申运河、芜湖港口、青弋江分洪道等项目建设，强化综合交通枢纽地位。

（三）深化改革开放，建设制度规范、行政透明、充满活力的创新型城市

着力创新体制机制，形成经济社会运行成本较低、效率较高、效益较好、以人为本、社会和谐、人民群众创造力得到充分发挥、更具活力、更加开放的制度环境。加快法治政府建设步伐，深化行政管理体制改革，促进政企分开、政资分开、政事分开、政府与市场中介组织分开，使芜湖成为行政审批项目少、环节短、收费低、规范透明的城市。推进事业单位分类改革，形成比较完善的社会事业管理体制。推进农村土地承包经营权流转制度改革，建立与户籍、社保、就业等制度改革相衔接的管理体制。深化国有资产管理体制改革，完善资产运营监管体制，确保国有资产保值增值。加快发展资本、产权、技术、人力资源等要素市场，建立比较完善的市场体系。推进社会信用体系建设，构建以道德为支撑、产权为基础、法律为保障的社会信用制度。全力推进招商引资，在招大商、引巨资上取得突破。加快转变外贸发展方式，立足以质取胜，优化出口产品结构，增强出口产品的国际竞争力。加强国内外经济技术交流与合作，实施“走出去”战略，鼓励有条件的企业拓展国际市场。

（四）加快推进以改善民生为重点的社会建设，努力构建和谐芜湖

基本建立覆盖城乡、比较完善的现代国民教育体系、社会保障体系、医疗卫生服务体系、社会管理体系。发展各级各类教育，推进全市中小学标准化建设，促进教育均衡发展，深化教育管理体制改革，鼓励社会资本投资教育，建设人力资源强市。实施扩大就业的发展战略，以创业带动就业。努力增加居民收入，建立企业职工工资正常调整机制和支付保障机制。以确保发放、扩大范围和完善制度为重点，加快建立覆盖城乡居民的社会保障体系。基本建立覆盖城乡的社会救助和灾害应急救助体系，完善社会福利体系和社会慈善事业发展机制。加强房地产市场监管，完善住房保障体系，调整住房供应结构，增加有效供给。继续推进拆迁安置房、廉租房、限价房建设，提高廉租房补贴标准，着力解决低收入家庭住房困难。完善基本医疗卫生制度，实行政事分开、管办分开、医药分开、营利性和非营利性分开，整合医疗卫生资源，改善医疗环境，建设覆盖城乡居民的医疗卫生服务体系、医疗保障体系、药品供应体系。重视社会组织建设和管理，健全党委领导、政府负责、社会协同、公众参与的社会管理格局。强化安全生产监管，完善突发事件应急管理体制，健全社会治安防控体系，推进平安芜湖建设。今后新增财力主要用于改善民生，按照“统筹规划、急事先办、尽力而为、务求实效”的原则，每年实施一批民生工程。

（五）推动文化大发展大繁荣，创建具有滨江山水园林特色的文化旅游名城

继续弘扬创新文化、和谐文化、廉政文化，用社会主义核心价值体系引领社会思潮，推动思想观念和体制机制创新，加强公民道德建设，打造芜湖城市精神，努力营造全社会创新创业的浓厚氛围，不断增强推动科学发展的凝聚力和向心力。加大文化事业投入，建成一批文化基础设施，形成比较完善的公共文化服务网络。深化文化体制改革，完善推进文化产业发展的政策体系，营造有利于出精品、出人才、出效益的社会环境。大力发展文化产业，引导社会投入，建成一批重大文化产业项目。把文化旅游产业作为新兴支柱产业着力加以培育，建设文化旅游目的地城市。以基础设施建设为重点，以动漫产业发展为抓手，加快华强旅游城建设步伐，打响文化旅游优质品牌。加强旅游景区及配套设施建设，拓展特色工业旅游项目。加强与周边地区旅游合作，优化精品线路的设计和组合。强化城市营销，提升城市知名度和美誉度。

三、努力实现新一届政府工作良好开局

2008年是新一届政府开局之年，也是实施“十一五”规划承上启下的关键一年，做好今年工作意义重大。今年经济社会发展的主要预期目标是：全市生产总值比上年增长15%以上，财政收入与经济同步增长，全社会固定资产投资增长25%，城市居民人均可支配收入增长10%，农村居民人均纯收入增长7%，城镇新增就业5万人以上，城镇登记失业率控制在4%以内，人口出生率控制在12‰以内，万元生产总值能耗下降4%，二氧化硫、化学需氧量分别减排1.4%和1%。

（一）推动产业结构优化升级

大力实施大企业大集团战略。出台支持大企业大集团发展的政策措施，支持骨干企业加快发展。建立培育大企业大集团联席会议制度，对大企业实施“直通车”服务。建立产业发展基金，重点支持企业研发投资。切实维护企业家合法权益，重奖贡献突出的企业领军人物，优化企业家成长环境。完善促进中小企业发展的政策体系，在信贷融资、技术支持、政策扶持等方面搞好服务，培育更多的市场主体。加快中小企业担保体系建设，发挥政策性担保公司的引导作用，着力解决中小企业融资困难。以示范企业、示范项目为重点，推广信息技术在企业管理、工艺、营销上的应用，加强“全国制造业信息化工程重点城市”建设，推进信息化与工业化融合。

大力发展现代服务业。推进芜申运河、芜雁高速、铜南宣高速、城际铁路和港口基础设施工程建设，完善交通枢纽功能。做好市域专业市场规划，划行归类，加快建设，集聚发展。推动吉和商业广场等一批大型商业项目建设。争取建设2个农产品批发市场和农产品出口示范基地。吸引域外金融机构在我市设立分支机构。积极发展地方金融机构，做大做强扬子农村商业银行，支持其在三县设立分支机构。筹建三县农村合作银行。争取成立奇瑞汽车金融公司。大力发展担保、典当、保险、信托、证券业，增强企业和个人的融资能力。创新金融产品，改进个人支付结算服务，提高非现金支付工具普及率。支持金融机构建设企业信用评级体系，改善金融生态环境。加快发展会计、律师、咨询等中介服务业，培育各类行业协会组织。政府支持，市场化运作，办好一批特色品牌会展。

加快发展都市型现代农业。开展特色农业“百村示范、千村带动”行动。大力发展无公害农产品、绿色食品、有机食品，培育地方特色农产品品牌。加强动植物疫病防控，提高农产品质量安全水平。支持一批农业产业化龙头企业做大做强。培育农民专业合作经济组织、农村经纪人和“执照农民”队伍。加速农业科技创新和科技成果转化，推广袁隆平超级稻“种三产四”丰产工程。实施农村劳动力转移培训“阳光工程”，扩大农村劳动力转移就业。强化农资市场管理，严肃查处制售假冒伪劣产品行为。

（二）着力转变经济发展方式

大力推进自主创新。推进我市“全国技术创新工程示范城市”工作，支持企业创建国家级和省级工程技术研究中心、企业技术中心、重点实验室。推动企业申报国家级、省级高新技术企业和高新技术产品。加快创业中心国家级企业孵化器和产业化基地建设步伐，芜湖汽车电子及关键零部件企业孵化器中试厂房建成投入使用，争取留学人员芜湖创业园建成国家级创业园。积极创建全国知识产权示范城市。

促进资源节约集约利用。加大土地开发复垦整理力度，确保耕地总量动态平衡。做好第二次土地调查。开展土地利用总体规划修编。严格林地征用管理。抓好城镇建设用地增加和农村建设用地减少挂钩试点，做好土地置换工作。制订工业用地管理办法，推行工业用地招拍挂出让，提高土地投资强度。加强长江岸线管理。整合矿山资源，关闭一批污染环境、有安全隐患的矿山。加强重点能耗企业监管，实施一批节地、节能、节水、节材和资源综合利用项目。

加强环境保护和生态市建设。提高环境保护门槛，严格执行新建项目环境影响评价制度和“三同时”制度。完善污染物排放许可制度，加强重点企业监控，实施污染物排放总量控制。加快淘汰落后产能，逐步关闭立窑水泥生产线。强化饮用水水源保护。重点整治涉铅、造纸、化工、电镀等行业企业，对环境污染突出的企业依法挂牌督办，限期整改。继续开展油烟、烟尘、噪声污染专项整治。实行环保问责制度，强化依法监管。完善节能减排考核办法，实行年终评优一票否决。

（三）加快统筹城乡发展步伐

今年市级将多渠道筹资50.5亿元，推进城乡基础设施建设。按照“东扩、南进、中提升”的思路，建设新区，改造老区，扇形拓展，加强主城区建设。推进城市东扩，加快三环路延伸段等道路建设，建成赤铸山东路、神山东路；加快商务文化中心、镜湖新城等项目建设步伐；继续整治神山公园；整合资源，启动一批东区教育、卫生等配套设施项目。加快老城区改造，扩建赭山公园，完成赭山路、长江路等道路“刚改柔” 和弋江路改造、滨江公园一期工程，继续推进保兴垾综合整治。完善城南路网体系，完成峨山路改造工程，新建滨江南路二期、龙湖路等主干道路。启动杨家门水厂扩建工程。推进污水、垃圾处理项目建设。扎实推进社会主义新农村建设。基本建成芜南路快速通道三期和芜南供水工程。继续推进万亩以上圩口堤防达标建设。完成14座小水库除险加固。加快永安桥等泵站建设。坚持“建养并重”，建设115公里乡级道路，改造桥梁65座，加强农村公路养护工作。继续实施村庄整治、农村户用沼气普及工程。强化城市管理，深入开展大拆违行动，健全户外广告有偿出让使用管理制度，加强城管数字化建设。改善公交服务，提升城市形象。完善城市分区规划、房地产及住宅建设规划，加强控制性详规、专业专项规划编制工作，强化日常监管。

（四）深入推进改革开放

深化农村改革，开展土地承包经营权流转与农村社保等制度改革挂钩试点。深化国有资产管理体制改革，促进企业股份制改造，加大企业直接融资力度，争取3家以上企业实现上市，2家已上市企业实现增资扩股，1家企业发行长期债券。完成全市事业单位分类改革。继续把招商引资作为经济工作的重中之重，紧抓不放。推进开发区二次创业，加快东区基础设施建设步伐，建成东区投资服务中心，吸引一批项目入驻。继续加大对县区发展的支持力度，完善县区工业园区功能，提高承载能力。创新招商方式，以小分队招商为主要形式，综合运用以商招商、产业招商等方式，吸引一批大项目落户，推动企业在芜建立研发中心、运营中心、财务结算中心。完善招商引资奖励办法，形成有效激励机制。支持企业“走出去”，开展跨国经营。扩大对外贸易，提高机电产品和高新技术产品的出口比重。

（五）发展文化事业和文化旅游产业

今年市级将筹资1.2亿元，支持文化基础设施建设和产业发展。加强爱国主义教育和公民道德建设，弘扬社会正气，引导公民自觉履行法定义务、社会责任、家庭责任。创建全国文明城市工作先进市，建设一批文明社区、文明村镇、文明单位。开展群众性文体活动，倡导科学、健康、文明的生活方式。以实施文化信息资源共享等工程为抓手，推进新农村文化建设。开展社会文化活动，举办市文化艺术节。建成市图书馆、文化馆、博物馆。开工建设市文化艺术中心。实施“振兴芜湖版画”等精品工程。加强对以铁画为代表的非物质文化遗产的保护、开发与利用。强化网络文化市场管理。加强档案、地方志工作，加快芜湖通史等修编，出版芜湖市志。鼓励社会资本投资文化产业，推动华强文化科技产业园等项目建设。建立动漫产业基金，加快建设国家级动漫产业基地。发展文化旅游产业，加强华强旅游城市场营销，加快芜湖古城、马仁奇峰、丫山花海石林、陶辛水韵等景区建设，完善配套设施，推动景区升级。

（六）大力推进社会建设

优先发展教育，今年市本级将筹资2.6亿元，用于教育基础设施建设。完善推进义务教育均衡发展制度与配套工作机制。全市义务教育阶段公办学校免收学杂费，对义务教育阶段农村家庭和城市困难家庭学生免收教科书费。在城乡推进普及三年学前教育。扩大优质高中资源。推进市职教园区、县级职教中心建设。鼓励社会投入，大力支持民办教育。

加强就业和社会保障工作。认真落实各项就业扶持政策，开展城乡统筹就业全国试点，加大小额担保贷款力度，推进全民创业。加快技师学院建设，加强职业技能培训。认真实施劳动合同法，保护劳动者权益。做好提高养老保险统筹层次基础性工作。统一城镇职工与农民合同制职工失业保险待遇。开展建立农村养老保险制度试点。强力推进“签约参保”，完善社会保险征缴工作机制。加强社会保险基金监管，建成“金保工程”。完善社会救助政策，保障困难群体、特殊群体、优抚群体的基本生活权益。

加强房地产市场监管，遏制房价过快上涨。加大房屋开发力度，增加住房用地供应量，加快中小户型、中低价位住房开发，竣工商品住房250万平方米以上。加大拆迁安置房建设力度，竣工拆迁安置房140万平方米，其中廉租房3万平方米。加强住房公积金监管，推动非公有制企业建立住房公积金制度。

切实缓解群众看病难、住院难问题。从今年开始，未来五年每年将投入不少于5000万元，整合医疗资源，支持医疗机构加快基础设施建设。加快市二院、市中医院、弋矶山医院、海螺医院门诊病房楼建设步伐，开工建设城东新区医院。推进农村医疗急救中心建设，完善城乡医疗急救网络。

加强社会管理。健全城乡社区民主管理制度，开展创建和谐社区活动。实施第七届村委会换届选举。强化价格监测和市场监管。加强人口和计划生育工作，改善计划生育技术服务条件，健全流动人口管理服务体系，稳定低生育水平。加强老龄工作。建立关注农村留守儿童工作机制，保障妇女儿童权益。加强国防后备力量建设，积极开展国防教育和双拥共建活动。加强信访工作。整合应急资源，推进应急平台建设，强化突发事件应急管理。落实安全生产责任制，着力整治安全隐患。加强社会治安综合治理，依法防范和打击违法犯罪活动，维护社会稳定。

（七）继续实施民生工程

今年继续筹资25.48亿元，实施24项民生工程：建立居民最低生活保障制度；提高农村“五保户”供养水平；完善城镇未参保集体企业退休人员基本生活保障机制；积极推进新型农村合作医疗制度；探索建立城镇居民基本医疗保障制度；逐步提高城乡医疗救助水平；逐步建立重大传染病病人医疗救治及生活救助保障机制；积极推进城乡医疗卫生服务体系建设；全面实施城乡义务教育经费保障机制改革；全面消除农村中小学现有D级危房；加速推进农村饮水安全工程建设；进一步完善农村部分计划生育家庭奖励扶助制度；实行库区移民补助机制；建立高职、中职在校家庭经济困难学生资助体系；实施白内障患者复明工程；实施广播电视村村通工程；实施廉租住房建设工程；实施农村公路村村通工程；实施食品药品安全工程；启动棚户区改造工程；推进市区环境综合整治工程；实施道路畅通工程；实施医药分开改革；建立统筹城乡就业困难人员托底就业机制。

（八）加强政府自身建设

坚持依法行政、民主决策，推进管理创新、制度创新，着力转变职能、提高效能，努力形成权责一致、分工合理、决策科学、执行顺畅、监督有力的行政管理体制，建设服务型政府。

着力推进法治政府建设。自觉接受人大及其常委会监督，认真落实人大决定，定期报告工作。积极支持人民政协履行政治协商、民主监督、参政议政职能，主动听取各民主党派、工商联、无党派人士、各人民团体的意见和建议。认真办理人大议案、代表建议和政协建议案、提案。严格按照法定权限和程序行使权力、履行职责，做到依法决策、依法办事。优化政府重大决策规则和程序，完善公众参与、专家咨询、政府决定相结合的行政决策机制。

着力推进制度建设。深入落实行政许可法，规范行政审批、许可行为。继续推进行政审批制度改革，完善“部门审批职能向一个科室集中、审批科室向行政服务中心集中”制度。深化公共预算改革，严格财政预算管理。完善政府招标采购管理体系，强化标后监管。加强政府绩效管理，加大对滥用权力和失职渎职行为的追究力度，实行行政问责。

着力推进管理创新。完善公共服务体系，推进电子政务建设。落实《政府信息公开条例》，大力推进政务公开，增强行政透明度。坚持从严治政，转变工作作风，大兴学习之风，改进会风、文风，力戒形式主义，切实提高政府公信力、执行力。

着力推进廉政建设。认真落实用人、用钱、用地、用权的各项制度，促进“四用”制度向公共权力运行的所有领域覆盖，向公共利益涉及的所有方面覆盖。建立行政审批电子监察系统，加强行政监察和审计监督。严格执行廉政建设责任制，深入治理商业贿赂，加大对违法违纪问题的查处力度，规范权力运行，全面推进惩治和预防腐败体系建设。

各位代表，发展蓝图已经绘就，新的征程任重道远，形势催人奋进，任务光荣艰巨。让我们在中共芜湖市委的坚强领导下，深入贯彻党的十七大精神，全面落实科学发展观，继续解放思想，坚持改革开放，推动科学发展，促进社会和谐，为芜湖全面建设小康社会、率先基本实现现代化而努力奋斗！
